# The Explanatory Effect of a Label: Its Influence on a Category Persists Even If We Forget the Label

**DOI:** 10.3389/fpsyg.2021.745586

**Published:** 2022-01-06

**Authors:** Ivan A. Aslanov, Yulia V. Sudorgina, Alexey A. Kotov

**Affiliations:** ^1^Faculty of Journalism, Lomonosov Moscow State University, Moscow, Russia; ^2^Laboratory for the Neurobiological Foundations of Cognitive Development, HSE University, Moscow, Russia

**Keywords:** label, explanation, memory, categorization, replication

## Abstract

In this study we replicated the explanatory effect of a label which had been found by Giffin et al. (2017). In their experiments, they used vignettes describing an odd behavior of a person based on culturally specific disorders that were unfamiliar to respondents. It turned out that explanations which explain an odd behavior through a person’s tendency to behave that way (*circulus vitiosus*) seemed more persuasive if the disorder was given a label that was used in the explanation. We replicated these results in Experiment 1, and in a follow-up Experiment 2 we examined the familiarity with category information and the evaluation of that category over time (the delay lasted one week). We realized that the label effect persists even when people make judgments based on their recollections about a category. Furthermore, according to a content analysis of the recollections, participants in the label condition remembered more information from the vignettes but tended to forget an artificial label; however, they used other words from the disorder domain instead (like “disease” or “kleptomania”). This allowed us to suggest a new interpretation of this effect: we suppose that in the Giffin et al. (2017) experiments the label did not bring any new features to a category itself, but pointed to a relevant domain instead, so the effect appeared from the activation of areas of knowledge in semantic memory and the application of relevant schema for learning a new phenomenon.

## Introduction

Labels are known to play an important role in categorization and category learning. According to a study by Gelman and Heyman (1999), using a noun label to refer to someone who has a certain property made children think of the property as more stable over time and in different situations. Another study by Yamauchi (2005) showed that using a categorical name as the title of a property of some person reinforced the tendency to make suggestions about other properties of that person. An extensive research series by Lupyan et al. (2007) describe the effects of category labels such as the ability to help adults to learn a category faster (Zettersten and Lupyan, 2020), to support mental representations that are more categorical (Lupyan, 2012) and prototypical (Lupyan, 2016), and to make knowledge more abstract, helping to go beyond concrete experiences and perceptions (Lupyan and Lewis, 2019).

Giffin et al. (2017) suggested that this property of categorical labels should affect the persuasiveness of explanations. In their experiments, they used vignettes describing culturally specific disorders unfamiliar to respondents. They found that explanations in which a sick person’s odd behavior was explained through that person’s tendency to behave that way seemed more persuasive if the disorder was given a label and that label was used in the explanation. The authors argued that named categories are more likely to license inferences to the presence of a cause that is responsible for what is being explained.

Believing that this effect in itself extends existing knowledge about the cognitive nature of explanations, we wanted to replicate the results of Giffin et al. (2017) and also to clarify whether the label has an effect not only on the evaluation of explanations, but also on the memory of information about the behavior to which the label was applied. Specifically, we wanted to see whether this effect would be revealed if respondents rated category statements not immediately after reading the vignette, but one week later. We also wanted to determine whether the label would have an effect on the amount of information remembered. Thus, we formulated two “empirically oriented” research questions:

RQ1: Would the explanatory effect of a label persist if respondents evaluated statements based on their memories of the stimulus material?

RQ2: Would the label affect the amount of information from the vignettes that respondents would remember? At the same time, the explanation offered by Giffin et al. (2017) seems rather general to us. The authors argue that “named categories lead people to draw an inference to the existence of a cause underlying the category, a cause that is responsible for the behavior being explained” (Giffin et al., 2017: 357). This does not explain why the presence of a label causes people to think there is an additional hidden cause; instead, it only declares that the label somehow points to the existence of such a cause. Furthermore, this explanation does not account for the social aspects of the categories used in the experiment, although the formation of a new category relating to human behavior involves prior social experience and theories about the human psyche (Cantor and Mischel, 1979).

A possible alternative explanation of the effect is presented in Hemmatian and Sloman (2018), in which the authors argue that “the explanatory value of labels arises in part from their ability to point to knowledge in the community” (Hemmatian and Sloman, 2018: 1689). By using a label, we induce people to believe that the community has additional knowledge about the phenomenon to be explained. This could add to Giffin et al.’s (2017) explanation, but it does not clarify why the label affects not only the persuasiveness of the explanation, but also the representation of multiple properties of the behavior being explained (such as the behavior’s stability over time, its psychological or biological nature, etc.). We hypothesized that the inclusion of a label in the description of an odd behavior affects the categorization itself: social experience tells people that mental disorders are accompanied by odd behavior and have specific names. Although the authors used artificial labels, even “meaningless” words can activate certain areas of semantic memory, prompting people to interpret these words based on their experiences (Davis et al., 2019). Additionally, some people tend to trust any information containing scientific terminology (Fernandez-Duque et al., 2015; Pennycook et al., 2015). Accordingly, the mere presence of a label may be a meaningful indication for categorizing the behavior being described as “disease,” and processing information according to that category’s schema — after all, according to the “theory of theories,” an individual’s social experience and naive theories can determine the outcome of categorization (Murphy and Medin, 1985; Heit, 1994; Murphy and Allopenna, 1994; Wisniewski and Medin, 1994; Lin and Murphy, 1997).

However, the importance of naive theories is evident not only in categorization and categorical learning, but also in explaining new information. For example, it is known that when constructing explanations, people tend to rely on information about the world that is available in their memory (Hussak and Cimpian, 2018). Other research shows that key attributes for category definition are remembered and recalled more easily than contextual attributes, and therefore explanation construction relies primarily on more typical attributes (Horne et al., 2019). Previous experiences available in memory are also involved in the formation of other types of judgments, such as hypotheses (Weber et al., 1993; Thomas et al., 2014).

If a label is needed only during categorization and plays the role of a marker of a relevant domain of knowledge, then in the future when a category is already formed, a person can make judgments about it even if the label is forgotten; the effects caused by the label will persist. Knowing that it is common for illnesses to provoke abnormal behavior and to have medical names, a person can treat the description of strange behavior as an example of illness, projecting the essential properties of the category “illness” onto that behavior, according to the theory of “principled connection” (Prasada and Dillingham, 2006). This is where our explanation differs from that of Giffin et al. (2017): we believe that the effect arises not because a label points to the existence of a cause underlying the category, but because it helps to present the new category as an example of an already existing category (in this case, “disease”) and to apply already existing knowledge to understanding the new information. Let us add that our explanation is different, but does not contradict the authors’ original explanation, and can be perceived as a clarification. Here we come to the third research question, focused on testing our assumption:

RQ3: Would participants in the experimental group (the label condition) talk about abnormal behavior as an illness more often than participants in the control group, when recalling the contents of the vignettes?

We replicated the label effect, and in a follow-up study used a time interval to separate the familiarity with category information and the evaluation of that category.

## Experiment 1 (Replication Study)

### Method

#### Participants

314 people participated in the replication (177 females, *M* = 21.82, *SD* = 7.31 years; 86% had incomplete higher education). 98 respondents were recruited online and were paid for participation (41 of them did not indicate age, but they were adults according to the terms of the recruiting website), and 216 participants were students of psychological courses who received extra credits for participation.

#### Materials

We used materials from the study by Giffin et al. (2017). The authors elaborated four vignettes, all representing a short story about a person with unusual behavior. Each vignette contained the description of a behavior with different features created, based on culture-specific disorders. The disorders were chosen due to their low probability of being commonly known among American people. Since we recruited Russian participants, there was also a low probability that the disorders would be familiar to them (the original material contained descriptions of disorders specific to certain regions of the world, such as Africa, Asia, and Latin America, and which are not found in Russia).

For each vignette, two versions were created. One included a category label for the described behavior, while the other did not contain any category label, and the behavior was described simply as a tendency. The category label was artificial (“depathapy”), and was the same in all vignettes. There were two vignettes about 40-year old men (David, Mark) and two about women (Laura, Maria) (see Table 1 for an example).

**TABLE 1:** An example of experimental material.

Category label condition	Control condition
“David is a 40-year-old male. Recently, he took a beautiful and expensive painting from his office after one of his co-workers said, “you should take that painting, you’re the only one who ever looks at it.” David’s co-worker had not been serious. *It turns out that David has Depathapy - a tendency* to imitate the actions of others and obey commands directed at them, leading him to take the painting.”	“David is a 40-year-old male. Recently, he took a beautiful and expensive painting from his office after one of his co-workers said, “you should take that painting, you’re the only one who ever looks at it.” David’s co-worker had not been serious. *It turns out that David has a tendency* to imitate the actions of others and obey commands directed at them, leading him to take the painting.”

*The differences between conditions and marked in italics.*

Fifteen statements were created for each vignette to evaluate participants’ judgments about the described behavior. Each statement was evaluated *via* a 7-point Likert scale, divided into blocks representing different aspects of behavior.

We adapted all materials for Russian speaking participants. Apart from translating the vignettes and statements in Russian, we changed three names from the vignettes to ones commonly known in Russia. The category label was literally translated as ДeΠaTaфия. Additionally, we used only thirteen statements instead of the original fifteen because of the inability to create a correct natural translation of two statements in Russian. Since there were no significant differences between conditions in these statements in the original study, we decided to remove them from the replication (see Table 2 for the list of statements). All materials, as well as data, are published online: https://osf.io/52dsn/.

**TABLE 2:** The list of the items.

Variable	Statement
**Block 1**	
Explanation	“Suppose someone asks why David took the painting. How satisfying do you find the following answer? ‘David acted this way because he has *Depathapy, a tendency/a tendency* to imitate the actions of others and obey commands directed at them’.” Rated on a scale of 1 (not at all satisfying) to 7 (very satisfying).
Blame	“How strongly would you agree or disagree that David deserves blame for taking the painting?” Rated on a scale of 1 (strongly disagree) to 7 (strongly agree).
Legal	“Suppose you are a juror in a court case trying David for his actions. The judge informs you that you should find David not guilty by reason of insanity if you believe that because of a mental disease or defect, he did not know or understand the nature and quality of his act or did not know or understand that his act was morally or legally wrong. How likely would you be to find David guilty?” Rated on a scale of 1 (not at all likely) to 7 (very likely).
**Block 2**	
Stability past	“Given David’s *Depathapy/tendency*, how likely do you think it is that he would have obeyed commands directed at him 5 years ago?” Rated on a scale of 1 (not at all likely) to 7 (very likely).
Stability future	“Given David’s *Depathapy/tendency*, how likely do you think it is that he might obey commands directed at him 5 years from now?” Rated on a scale of 1 (not at all likely) to 7 (very likely).
Generalize others	“How likely is another person with *Depathapy/tendency* to exhibit behavior resulting from a tendency to imitate the actions of others and obey commands directed at them, similar to that exhibited by David (when in a similar position)?” Rated on a scale of 1 (not at all likely) to 7 (very likely).
Generalize self	“How likely would you be, in David’s position, to exhibit behavior resulting from a tendency to imitate the actions of others and obey commands directed at you, similar to that exhibited by David?” Rated on a scale of 1 (not at all likely) to 7 (very likely).
**Block 3**	
Text before statements from Block 3	“David’s *Depathapy/tendency* could be caused by biological or psychological factors. Biological factors include any genetic or physiological factors that contribute to or cause the condition. Psychological factors include any behaviors, thoughts, emotions, or identity-related factors that contribute to or cause the condition.”
Biological	“To what extent is David’s *Depathapy/tendency* BIOLOGICAL in nature?” Rated on a scale of from 1 (not at all) to 7 (completely/entirely).
Psychological	“To what extent is David’s *Depathapy/tendency* PSYCHOLOGICAL in nature?” Rated on a scale of from 1 (not at all) to 7 (completely/entirely).
**Block 4**	
Text before statements from Block 4	“David’s *Depathapy/tendency* could be treated by either medication or psychotherapy. Medication refers to any psychiatric, psychoactive, or psychotropic drugs. Psychotherapy refers to treatment by psychological means, involving repeated verbal interactions between a clinician and a client.”
Medication	“To what extent could David’s *Depathapy/tendency* be improved, controlled, or managed by medication?” Rated on a scale of 1 (not at all) to 7 (very effectively).
Therapy	“To what extent could David’s *Depathapy/tendency* be improved, controlled, or managed by psychotherapy?” Rated on a scale of 1 (not at all) Дo 7 (very effectively).
**Block 5**	
Common cause	“How strongly do you agree or disagree with the idea that there is a common cause that is shared by all and only people with *Depathapy/tendency* (whether or not we know what that cause is)?” Rated on a scale of 1 (strongly disagree) to 7 (strongly agree).
Common symptoms	“How strongly do you agree or disagree with the idea that there are common symptoms shared by all and only people with *Depathapy/tendency* (whether or not we know what all these symptoms are)?” Rated on a scale of 1 (strongly disagree) to 7 (strongly agree).

*The list of statements for one vignette. The differences between conditions are written via slash and marked in italics.*

#### Procedure

The experiment was conducted online. Participants were randomly assigned to one of four vignettes and one of two conditions: with or without a category label. First, participants saw a vignette on the screen and were asked to read it carefully. Then they were asked to evaluate thirteen statements using a 7-point Likert scale. The statements were divided into five blocks, with each block presented on a single screen. After the statement’s evaluation, participants provided their demographic information (age, gender, education level). The materials and questionnaire are available online: https://osf.io/n6h8y/.

### Results

Explanations from the category label condition were evaluated as more satisfying than those in the control condition: *F*(1,306) = 10.30, *p* = 0.001, η_p_^2^ = 0.033. In particular, participants gave higher scores for the statement: “…[name] did it because he/she has *depathapy, a tendency to*…” in comparison with the scores given by participants from the control condition who were presented with the following explanation: “…[name] did it because he/she has *a tendency to*…”. See Table 3 for descriptive statistics.

**TABLE 3:** Descriptive statistics.

	Giffin et al. (2017) study		Experiment 1		Experiment 2	
	Category label	Control (without label)	Category label, *n* = 168	Control, *n* = 146	Category label, *n* = 68	Control, *n* = 65
Explanation	**4.40 (1.80)**	**3.40 (1.93)**	4.97 (1.93)**	**4.32 (1.76)**	4.91 (1.70)	4.83 (1.76)
Blame	**4.55 (1.69)**	**5.40 (1.67)**	3.56 (1.90)***	**4.56 (1.76)**	**3.49 (1.66)** ^	**3.94 (1.65)**
Legal	4.06 (1.93)	4.23 (1.77)	3.19 (1.93)**	**3.89 (1.72)**	2.76 (1.56)	2.98 (1.68)
Stability	5.57 (1.12)	5.55 (1.05)	5.11 (1.17)	5.01 (1.29)	5.01 (1.29)	5.17 (1.29)
Generalization to others	**5.31 (1.37)**	**4.29 (1.56)**	5.45 (1.42)***	**4.21 (1.88)**	5.57 (1.18)**	**4.02 (1.47)**
Generalization to self	**4.28 (1.93)**	**2.94 (1.81)**	4.33 (2.03)***	**2.75 (1.89)**	4.93 (1.58)**	**3.26 (1.88)**
Biological	**4.08 (1.52)**	**3.50 (1.53)**	4.18 (1.34)*	**3.75 (1.58)**	4.63 (1.16)	4.12 (1.51)
Psychological	**4.78 (1.39)**	**5.28 (1.26)**	5.03 (1.30)*	**5.46 (1.31)**	**4.84 (1.22)^**	**5.49 (1.21)**
Medication	4.69 (1.47)	4.74 (1.33)	5.00 (1.36)**	**4.47 (1.47)**	4.93 (1.36)	4.55 (1.52)
Therapy	4.88 (1.35)	5.14 (1.29)	5.26 (1.34)	5.33 (1.31)	5.12 (1.22)	5.68 (1.00)
Common cause	**4.21 (1.38)**	**3.73 (1.43)**	4.15 (1.47)	4.07 (1.59)	4.38 (1.35)	4.42 (1.30)
Common symptoms	**4.71 (1.58)**	**4.11 (1.46)**	5.13 (1.25)	4.90 (1.24)	5.34 (1.03)	5.17 (1.01)

Means and standard deviations for each statement in the original study, Experiment 1 and Experiment 2. Values with significant differences between experimental and control conditions are marked bold. *** - p < 0.0001, ** - p < 0.001, * - p < 0.01, ^ - p < 0.05.

#### Blame

Participants from the category condition tended to blame the person for his or her actions less than participants from the control condition: *F*(1,306) = 26.63, *p* < 0.0001, η_p_^2^ = 0.080. This is similar to Giffin et al.’ (2017) study (η^2^ = 0.071).

#### Legal

Participants from the category label condition were less likely to think the person had to be punished for his or her actions than those from the control condition: *F*(1,306) = 11.98, *p* < 0.001, η_p_^2^ = 0.038. The effect of the label was not found in the original study.

#### Stability

Following Giffin et al.’ (2017) study, we averaged answers on statements about the stability of the behavior in the past and in the future. There was no significant effect of the category label: *F*(1,306) = 0.495, *p* = 0.48, η_p_^2^ = 0.002.

#### Generalization to Others

Participants from the category label condition were more likely to think that other people would demonstrate the same behavior as the person from the vignette, compared with participants from the control condition: *F*(1,306) = 48.31, *p* < 0.0001, η_p_^2^ = 0.136. These results are consistent with the original study (η^2^ = 0.111).

#### Generalization to Self

We found a significant effect of the category label: *F*(1,306) = 53.21, *p* < 0.0001, η_p_^2^ = 0.148. Participants from the category label condition were more likely to think they would exhibit the same behavior as the person from the vignette, compared with participants from the control condition, which is consistent with Giffin et al.’ (2017) study, (η^2^ = 0.120).

#### Biological Nature

Compared with the control condition, participants from the category label condition were more likely to believe in the biological nature of the behavior: *F*(1,306) = 7.32, *p* < 0.01, η_p_^2^ = 0.023. This is similar to what Giffin et al. (2017) found in their study (η^2^ = 0.036). However, we also found significant factor interactions: *F*(3,306) = 2.94, *p* = 0.03, η_p_^2^ = 0.028.

#### Psychological Nature

Participants from the category label condition were less likely to believe the described behavior to be psychological in nature: *F*(1,306) = 8.47, *p* < 0.01, η_p_^2^ = 0.027. This result is similar to the original study, (η_p_^2^ = 0.037).

#### Medication

Participants from the category label condition were more likely to agree that the behavior can be treated or controlled with medication, compared with those from the control condition: *F*(1,306) = 11.06, *p* < 0.001, η_p_^2^ = 0.035. These results differ from the original study, in which there were no significant differences between conditions.

#### Therapy

Participants from both conditions did not differ in their judgments that the behavior can be controlled or improved using psychotherapy: *F*(1,306) = 0.24, *p* = 0.62, η_p_^2^ = 0.001.

#### Common Cause

Participants from the category label and control conditions were not different in their judgment about a common cause of the behavior: *F*(1,306) = 0.22, *p* = 0.64, η_p_^2^ = 0.001. Participants from Giffin et al.’ (2017) study gave significantly different answers (η^2^ = 0.031).

#### Common Symptoms

Participants did not differ in their judgments about common symptoms of the behavior, depending on the condition to which they were assigned: *F*(1,306) = 2.58, *p* = 0.1, η_p_^2^ = 0.008. An effect of the category label was found in the original study (η^2^ = 0.040).

ANOVA also revealed a significant factor of the vignette in some statements. Similar to Giffin et al.’ (2017) study, we found a significant effect of the vignette in the following statements: Blame: *F*(3,306) = 16.70, *p* < 0.0001, η_p_^2^ = 0.141 (η^2^ = 0.129); Legal: *F*(3,306) = 5.97, *p* < 0.001, η_p_^2^ = 0.055 (η^2^ = 0.123); and Stability: *F*(3,306) = 3.79, *p* = 0.01, η_p_^2^ = 0.036, (η^2^ = 0.073).

Additionally, our analysis discovered an effect of vignette in Explanation: *F*(3,306) = 7.37, *p* < 0.0001, η_p_^2^ = 0.067; Generalization to others: *F*(3,306) = 4.99, *p* = 0.02, η_p_^2^ = 0.089; Generalization to self: *F*(3,306) = 7.27, *p* < 0.0001, η_p_^2^ = 0.067; and Medication statements: *F*(31,306) = 4.49, *p* < 0.01, η_p_^2^ = 0.042.

The results also revealed an interaction of both factors in the following statements: Explanation: *F*(3,306) = 3.20, *p* = 0.02, η_p_^2^ = 0.030; Generalization to others: *F*(3,306) = 9.96, *p* < 0.0001, η_p_^2^ = 0.047; Biological nature: *F*(3,306) = 2.94, *p* = 0.03, η_p_^2^ = 0.028; and Psychological nature: *F*(3,306) = 2.88, *p* = 0.03, η_p_^2^ = 0.027.

### Discussion

We repeated the study by Giffin et al. (2017) and replicated the explanatory effect of a label. We obtained significant differences in 7 of the 9 statements in which differences were reported by the original study’s authors (except Common cause and Common symptoms). We also obtained differences in one question in which the authors did not find differences (Legal). Overall, the label effect was replicated, and it was possible to use the experimental materials to examine the association of time delay with the label effect.

## Experiment 2

### Method

#### Participants

133 subjects took part in the experiment (117 female; *M* = 21.1, *SD* = 4.5 years). They were students from psychological courses and were given extra credits for participation. None of the participants participated in the Experiment 1.

#### Materials

We used the same materials as in the Experiment 1.

#### Procedure

The experiment was conducted online and consisted of two phases with a one-week interval between them. The materials and questionnaire are available online: https://osf.io/n6h8y/.

##### Phase 1

Participants were randomly assigned to one of four vignettes and one of two conditions. They were instructed that in the first part of the study they would read a vignette. They were informed that the second part of the study would be connected with the vignette so they were asked to read it carefully and to understand its content, but they were not aware of what exactly would be tested in the second phase After reading it, they answered a few questions about the vignette and completed demographic information. The web link to this part of the study worked for only 24 h to make sure all participants completed the first phase in one day.

##### Phase 2

Seven days later, we sent links for the study’s second phase. Depending on which vignette and in which condition the subjects participated previously, we sent a particular link with statements appropriate for certain versions of the vignette. Participants were instructed that they would evaluate some statements about the vignette they read a week before. First they were asked to recall the vignette in detail and write it in the response field. Then they were redirected to a questionnaire where they evaluated thirteen statements. They could not return to their written text and change it after the statements’ evaluation. Similar to the first phase, this was available for only 24 h. All students who participated in phase 1 continued their participation in phase 2.

#### Coding

As previously mentioned, participants were asked to recall the vignettes before answering the questions. The recollections of the participants were coded into two categories: (1) illness, and (2) non-illness. Responses were categorized as “illness” if they used the word “illness,” its synonyms, or the name of a real disease (i.e., “kleptomania”) to name the described tendency of a behavior. The other responses were categorized as “non-illness.” We also made lists of facts that were mentioned in each vignette and checked how many of these facts were mentioned in each response. The name of the described person, their age, other actors (like “colleague” or “police”), and their actions (like “took a painting” or “took her into custody”), as well as the symptoms of the diseases (like “to imitate the actions of others”), were considered as “facts.” Considering that the vignettes contained different numbers of facts (three vignettes contained 10 facts, but 1 vignette contained 9 facts), we weighed the number of facts in each answer against the number of facts in the corresponding vignette.

### Results

#### Statistical Analysis

Only significant results are reported below.

##### Blame

Participants from the category label condition were less likely to blame the person for his or her behavior, compared with participants from the control condition: *F*(1,125) = 4.47, *p* = 0.037, η_p_^2^ = 0.035.

##### Generalization to Others

We found the category label to be a significant factor in judgments about the probability that other people would behave the same way in the situation described in the vignette. Participants from the category label condition believed in this probability more than the ones from the control group: *F*(1,125) = 42.05, *p* < 0.001, η_p_^2^ = 0.252.

##### Generalization to Self

The effect of the category label was the same as in the previous statement—participants from the category label condition were more likely to think they would exhibit the same behavior as the person from the vignette than participants from the control condition: *F*(1,125) = 30.95, *p* < 0.001, η_p_^2^ = 0.198.

##### Psychological Nature

Participants from the category label group were less likely to believe in the psychological nature of the behavior, compared with those from the control condition: *F*(1,125) = 6.157, *p* = 0.014, η_p_^2^ = 0.047.

Apart from the category label factor, the vignette factor was also significant in the statements about Explanation: *F*(3,125) = 2.650, *p* = 0.05, η_p_^2^ = 0.060; Blame: *F*(3,125) = 7.29, *p* < 0.001, η_p_^2^ = 0.149; Biological nature: *F*(3,125) = 3.07, *p* < 0.05, η_p_^2^ = 0.059; Medication: *F*(3,125) = 4.35, *p* < 0.01, η_p_^2^ = 0.082; and Therapy: *F*(3,125) = 4.22, *p* = 0.007, η_p_^2^ = 0.092. Moreover, there was an interaction of condition and vignette factors in the following statements—Blame: *F*(3,125) = 3.59, *p* = 0.016, η_p_^2^ = 0.079; Stability: *F*(3,125) = 3.161, *p* = 0.027, η_p_^2^ = 0.071; Generalization to others: *F*(3,125) = 7.37, *p* = 0.005, η_p_^2^ = 0.098; Therapy: *F*(3,125) = 2.75, *p* = 0.046, η_p_^2^ = 0.062.

We planned to compare similar conditions (i.e., category label conditions and control conditions) from Experiment 1 and Experiment 2 in order to check for any differences between them. To make such a comparison, we first needed to find the interaction between the Experiment and Condition factors. The ANOVA did not reveal an interaction of the factors in any of the statements, *p* > 0.1 (except in *Therapy p* = 0.064).

To make sure that there was no interaction between factors in the Explanation statement, we ran a power analysis using G*Power which was equal to 0.319. We also used Bayesian ANOVA for the same statement to verify the absence of any interaction between factors, *BF*_10_ = 0.263, indicated moderate support for the null hypothesis of no interaction over the alternative hypothesis of an interaction. This means that we did not find changes in judgment in Experiment 2 compared to Experiment 1 in the label and non-label conditions. This also means that where significant differences were found in Experiment 1 but not in Experiment 2 (*Explanation, Legal, Stability, Biological nature*) we have little evidence that these differences are in fact absent in Experiment 2.

Additionally, we checked for the influence of the condition factor in both experiments simultaneously. ANOVA did not reveal any differences in Stability, Common cause and Common Symptoms (*p* > 0.1). However, there were significant differences in Explanation (*p* = 0.054), Blame (*p* < 0.001), Legal (*p* = 0.012), Generalization to others and Generalization to self (both *p* < 0.001), Biological nature (*p* = 0.001), Psychological nature (*p* < 0.001), Medication (*p* = 0.002), and Therapy (*p* = 0.016). Thus, the results demonstrate the presence of the label effect.

#### Coding Results

Overall, participants were more likely to categorize the strange behavior as “illness” in the label condition (72%) than in the control condition (25%): χ^2^(1) = 29.9, *p* < 0.001, *N* = 133. Only 5 out of 68 participants in the label condition mentioned the label “depathapy.” They tended to use some labels from the “illness” domain instead, such as “mental disorder,” “disease,” “psychological pathology,” “mental sickness,” “phobia,” and “kleptomania.”

We used Student’s *t*-test to analyze the differences in named facts between conditions. As predicted, the rate of named facts was also significantly higher [*t*(131) = 2.85, *p* = 0.005] in the label condition (*M* = 0.37, *SD* = 0.23) than in the control condition (*M* = 0.27, *SD* = 0.19). The test showed that the numbers of named facts were significantly lower than the number of facts in the vignettes in both experimental [*t*(67) = −23.5, *p* < 0.001] and control groups [*t*(64) = -31.3, *p* < 0.001].

### Discussion

We modified the replicated experiment by interviewing respondents not immediately, but one week after reading the stimulus material. We found that the label affected at least 4 questions, and in those statements where the effect was found in Experiment 1 but not in Experiment 2, the effect size did not differ. The content analysis showed that the label helps readers to better remember information about the new category. Also, in the case of the label, respondents were more likely to call the new phenomenon a disease, while the label “depathapy” itself was forgotten by almost all participants.

## General Discussion

We replicated the results of the Giffin et al. (2017) experiment, showing that a label reinforces the perceived objectivity of a category. In addition, we were able to show that the label effect persists over time (RQ1), and that the labeled category retains the influence of the label even when the label itself is forgotten. We have also shown that having a label to describe an abnormal behavior helps respondents to remember more information about that behavior (RQ2).

According to our content analysis, the label also influenced the fact that respondents, when recalling descriptions of the strange behavior, tended to call it a “disease” or even to use the names of other diseases they knew instead of the artificial label (RQ3). In our view, this supports our assumption that in this experiment the label does not simply indicate the existence of a cause, as Giffin et al. (2017) claimed, but makes one think that “*depathapy*” *is a disease*. Not surprisingly, in thinking of depathapy as an example of a disease, people evaluate judgments about it based on the “principled connection” between the category “disease” and its kind, as described by Prasada and Dillingham (2006). Therefore, in evaluating the persuasiveness of the explanation, as well as other properties of “depathapy,” people refer to their existing knowledge from the disease domain, rather than simply assuming the existence of some cause or additional knowledge in the community, as suggested by Hemmatian and Sloman (2018). Although factors such as the causality and conventionality of the label still seem to play a role, the label’s ability to point to a relevant area of knowledge is also important. If this were not the case, we would see that the influence of the label on judgment evaluation persist, but people do not talk about “depathapy” in terms of illness. In fact, we can assume that having a label for an odd behavior simply increases the likelihood that it will be categorized as a disease (because people know that diseases have scientific names). So the label is indeed a kind of pointer, not to an abstract cause’ (as Giffin et al., 2017 suggest), but to a relevant concept/domain, which in turn may indeed contain additional information about both causality and other relevant properties.

Here we can highlight two questions which may be clarified in further research. Would an effect similar to the label effect arise if the domain was clarified by social context—for example, if a person saw two doctors discussing strange behavior with each other (but without a label)? Also, would there be an effect similar to the label effect if unlabeled information were shown to people whose theory of the world is not naive in the health domain (e.g., doctors)? As we know, expertise in a domain can have an effect on categorical proceedings and judgments (Proffitt et al., 2000; Hayes and Chen, 2008; Wattenmaker et al., 2015). Furthermore, there is already some evidence that medical professionals are also influenced by artificial labels that are claimed to be conventional when it comes to explaining illnesses (Hemmatian et al., 2019). Nevertheless, it would be interesting to discover whether such a label encourages the average person to look at abnormal behavior in a manner that is typical of medical professionals by default. Finally, mixed design examinations of vignette effects could be used to find out which aspect of the scenarios is having an unintended effect and whether that undermines the main findings.

Each type of label manipulation mentioned above can be important, especially in light of existing experiments that involve such manipulations and show that the principled connections theory cannot fully explain their consequences. In particular, Hemmatian and Sloman (2018) show that a label is a required condition for an uninformative category to appear as informative (see Experiment 4). It is necessary to find a theoretical explanation that reconciles the results of our experiment with the results of the mentioned study.

We would also like to point out the main limitations of our study: (1) The specific domain of the category, namely disease and human behavior, is only a single domain. Perhaps in the future it will be necessary to test this effect in categories of other domains. (2) Participants in the experiment replication group did not retell the text of the vignette, although participants in the main experiment group did. Perhaps in the future it will be worthwhile to achieve more equality between the two groups being compared.

Thus, we hope that our findings can clarify the mechanism of the explanatory effect of a label and can point the way for further research into this phenomenon.

## Data Availability Statement

The datasets presented in this study can be found in online repositories. The names of the repository/repositories and accession number(s) can be found below: https://osf.io/52dsn/.

## Ethics Statement

Ethical review and approval was not required for the study on human participants in accordance with the local legislation and institutional requirements. Written informed consent for participation was not required for this study in accordance with the national legislation and the institutional requirements.

## Author Contributions

All authors listed have made a substantial, direct, and intellectual contribution to the work, and approved it for publication.

## Conflict of Interest

The authors declare that the research was conducted in the absence of any commercial or financial relationships that could be construed as a potential conflict of interest.

## Publisher’s Note

All claims expressed in this article are solely those of the authors and do not necessarily represent those of their affiliated organizations, or those of the publisher, the editors and the reviewers. Any product that may be evaluated in this article, or claim that may be made by its manufacturer, is not guaranteed or endorsed by the publisher.
